# Defining cutaneous molecular pathobiology of arsenicals using phenylarsine oxide as a prototype

**DOI:** 10.1038/srep34865

**Published:** 2016-10-11

**Authors:** Ritesh K. Srivastava, Changzhao Li, Zhiping Weng, Anupam Agarwal, Craig A. Elmets, Farrukh Afaq, Mohammad Athar

**Affiliations:** 1 Department of Dermatology and Skin Diseases Research Center, University of Alabama at Birmingham, Alabama, USA; 2 Division of Nephrology, Department of Medicine, School of Medicine, University of Alabama at Birmingham, Alabama, USA

## Abstract

Arsenicals are painful, inflammatory and blistering causing agents developed as chemical weapons in World War I/II. However, their large stockpiles still exist posing threat to public health. Phenylarsine oxide (PAO), a strong oxidant and a prototype arsenical is tested for its suitability to defining molecular mechanisms underlying arsenicals-mediated tissue injury. Topically applied PAO induces cutaneous erythema, edema and micro-blisters. These gross inflammatory responses were accompanied by the enhanced production of pro-inflammatory cytokines, ROS and unfolded protein response (UPR) signaling activation. To demonstrate the involvement of UPR in the pathobiology of these lesions, we employed chemical chaperone, 4-phenylbutyric acid (4-PBA) which attenuates UPR. 4-PBA significantly reduced PAO-induced inflammation and blistering. Similar to its effects in murine epidermis, a dose- and time-dependent upregulation of ROS, cytokines, UPR proteins (GRP78, p-PERK, p-eIF2α, ATF4 and CHOP) and apoptosis were observed in PAO-treated human skin keratinocytes NHEK and HaCaT. In addition, 4-PBA significantly restored these molecular alterations in these cells. Employing RNA interference (RNAi)-based approaches, CHOP was found to be a key regulator of these responses. These effects are similar to those manifested by lewisite suggesting that PAO could be used as a prototype of arsenicals to define the molecular pathogenesis of chemical injury.

Vesicants are a group of chemicals that cause skin blistering and inflammation following their cutaneous exposure^1^,^2^,^3^. One such class of agents is known as arsenicals^4^. Considering the feasibility of their synthesis and potential for instant painful tissue injury in humans, arsenicals were developed as chemical weapons to be used during the World War I/II^5^,^6^,^7^. The examples of arsenicals synthesized during this period include lewisite (L), methyldichloroarsine (MD), phenyldichloroarsine (PD), and ethyldichloroarsine (ED) besides others. Lewisite, while being highly toxic among these became the lead candidate for ammunition^8^,^9^. Large quantities of lewisite were manufactured by countries like Germany, Italy, USA, Soviet Union and Japan^7^,^10^,^11^. Although chemical warfare agents are discontinued to be developed by many nations, stockpiles of lewisite are still known to be present in many countries^12^,^13^. Therefore, accidental or intentional exposure to these arsenicals or/and their degradation products is still considered possible. One such example is the exposure of residents of Kamisu, Japan to diphenylarsinic acid (DPAA), which is a degradation product of diphenylchloroarsine (Clark I) or diphenylcyanoarsine (Clark II)^14^. The exposure to DPAA contaminated drinking water caused neuronal syndrome with cerebellar symptoms which initially became apparent among Kamisu residents^14^. Therefore, understanding the molecular pathobiology associated with the exposure to these chemicals warrant further in-depth investigation that may ultimately define molecular mechanism of action of these arsenicals and their intervention.

Lewisite can easily be mixed with other warfare agents such as sulfur mustard to augments its toxic effects^15^,^16^. Lewisite exposure produces irritation, redness, edema and blistering of the skin^16^,^17^,^18^,^19^,^20^. Systemic toxicity of lewisite following its cutaneous exposure has also been reported in humans and experimental animals^8^,^21^,^22^,^23^. In earlier studies, it was shown that reaction of arsenicals with cellular glutathione leads to protein thiol loss. Dysregulation of calcium homeostasis leading to oxidative stress, lipid peroxidation membrane damage and cell death was also reported to occur following lewisite exposure. Sulfhydryl groups on enzymes were shown to be the potential targets for the interaction with lewisite and possibly with other similar chemicals leading to the inhibition of their activities^24^,^25^. However, the exact molecular mechanism by which these arsenicals contribute to pathobiology of skin injury remains undefined.

Currently, these agents are classified as restrict agents and their access in the USA is only allowed to agencies with adequate facilities for safe storage, handling, use and decontamination of these chemicals. Therefore, planning and conducting multiple mechanistic studies with the parent arsenicals is difficult and expensive. While we are undertaking mechanistic studies following exposure to these arsenicals with MRIGlobal (Kansas City, MO), we found a relatively less toxic arsenical ‘phenylarsine oxide (PAO)’ which could be procured from Sigma (St. Louis, MO) for the laboratory use following required institutional approvals.

PAO is reported to be an analog of lewisite and other similar chemicals, which is also a chemical warfare degradation product (CWDP)^26^,^27^,^28^. Similar to other arsenicals, PAO is a membrane-permeable trivalent arsenical that specifically complexes with vicinal sulfhydryl groups of proteins to form stable ring structures^29^,^30^,^31^. It also binds with thiol groups and inhibits the action of many thiol containing enzymes^29^,^32^. Thus, it closely shares biochemical reaction profile with lewisite and many other similar arsenicals. Based on these biochemical similarities, we considered it as a surrogate of arsenicals which may manifest many pathobiological effects identical to those reported for other more dangerous analogs *albeit* with reduced severity.

In this study, we employed PAO to define the molecular mechanism underlying pathobiology of arsenicals. Our data show that cutaneous exposure to PAO in Ptch1^+/−^/SKH-1 mice causes intense inflammatory and tissue damaging responses. These effects are similar but potentially less severe than those reported to be manifested by lewisite and other similar more reactive arsenicals^17^,^28^,^33^,^34^. Our studies demonstrate that the pathogenesis of arsenicals-induced cutaneous injury is mediated by the activation of UPR signaling pathway.

## Results

### Single topical application of PAO induces severe cutaneous inflammation and micro-blistering in Ptch1^+/−^/SKH-1 hairless mice

**Figure 1:**
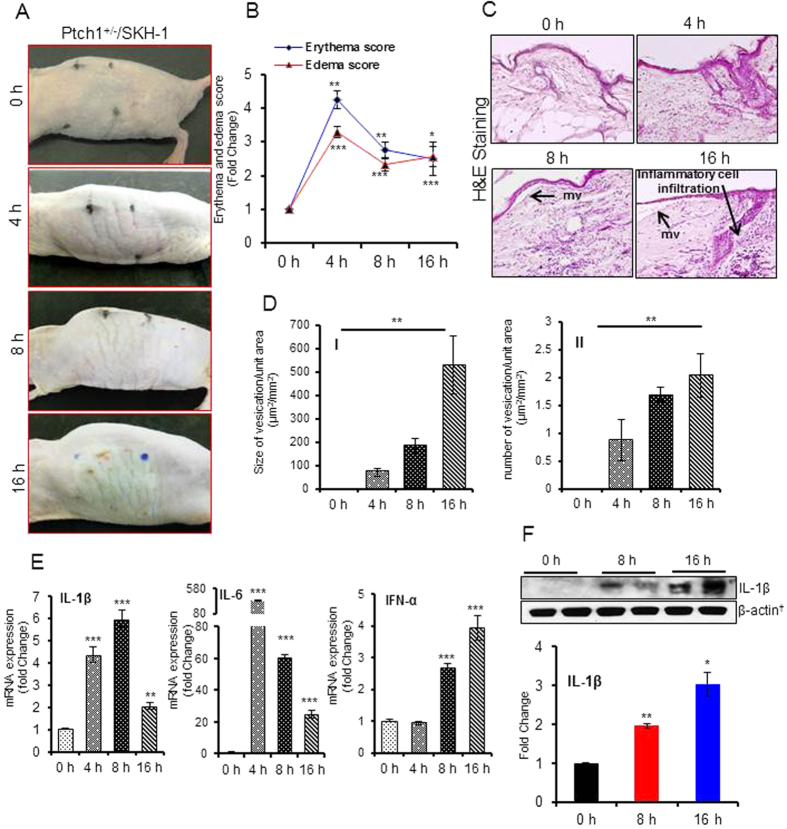
Topical application of PAO induces erythema, edema, micro-vesicants (mv) and inflammatory cytokines in Ptch1^+/−^/SKH-1 mouse skin. Dorsal skin of mice was treated topically with either 30 μL of ethanol (vehicle) or PAO (100 μg/mouse) in 30 μL ethanol on the dorsal skin area of 1.6 × 1.6 cm^2^ and animals were observed for PAO-induced effects/lesions at 0–16 h. (**A**) Mouse skin photographs showing time-dependent gross changes. (**B**) Effects of PAO on the development of erythema and edema using draize score as described in Supplementary Table S1. (**C**) Histochemical analysis of the skin sections (H&E) excised at various time intervals (0–16 h) following PAO treatment. Arrows show mv formation characterized by epidermal and dermal separation. (**D**) Time-dependent effects of PAO on size and number (**D**-**II**) of mv formation. (**E**) Histograms showing real time PCR analysis of pro-inflammatory cytokines. (**F**) Western blot analysis of IL-1β. Histogram showing dentiometric analysis of western blots. Data are expressed as mean ± SEM. *P < 0.05, **P < 0.01 and ***P < 0.001 when compared to controls (0 h).

Ptch1^+/−^/SKH-1 is highly sensitive murine model developed in our laboratory to demonstrate the toxic manifestations of environmental agents on the skin^35^. Selection of Ptch1^+/−^/SKH-1 hairless mouse strain employed in this study was based on the effects of PAO on cutaneous injury (erythema and edema) in various mouse strains such as FVB, C57BL/6, SKH-1 and Ptch1^+/−^/SKH-1^36^. Single application of PAO to Ptch1^+/−^/SKH-1 mouse skin caused marked redness within 45 min which became more pronounced with the progression of time. At 4 h, a significant enhancement in erythema was observed. Then the skin became thick, leathery, and wrinkled. At later time-points (8 h and 16 h) it turned into greyish lesion (Fig. 1A). The erythema and edema data were compiled using draize score as shown in Supplementary Table S1. The draize scores reveal a time-dependent enhancement in the cutaneous injury. At early time interval up to 4 h these values maximized, followed a slight decrease in these values at 8 h and 16 h (Fig. 1B). The histopathological analysis of H&E stained skin sections from age-matched vehicle-treated controls (0 h) and PAO-treated animals are shown in Fig. 1C. PAO treatment resulted in the disruption of cutaneous architecture in a time-dependent manner. Under the microscope, we observed a time-dependent increase in both size and number of micro-vesicants (mv) formation in the skin (Fig. 1D). The mv was characterized by the epidermal and dermal separation (Fig. 1C). We also observed a huge infiltration of inflammatory leukocytes in the dermal region surrounding these lesions (Fig. 1C). The inflammatory reaction was further characterized by the release of pro-inflammatory cytokines. The real time PCR analysis showed significant up-regulation of pro-inflammatory cytokines IL-1β, IL-6 and IFN-α in PAO-treated skin (Fig. 1E), which was further confirmed by western blot analysis (Fig. 1F). Interestingly, these responses are similar to lewisite-mediated cutaneous inflammation and tissue injury^36^. However, unlike other vesicants such as mustards which show a significant increase in TNF-α^37^, we observed a significant reduction in TNF-α at these time intervals (Supplementary Fig. S1).

### PAO-induced cutaneous damage is associated with ROS generation and apoptosis

**Figure 2:**
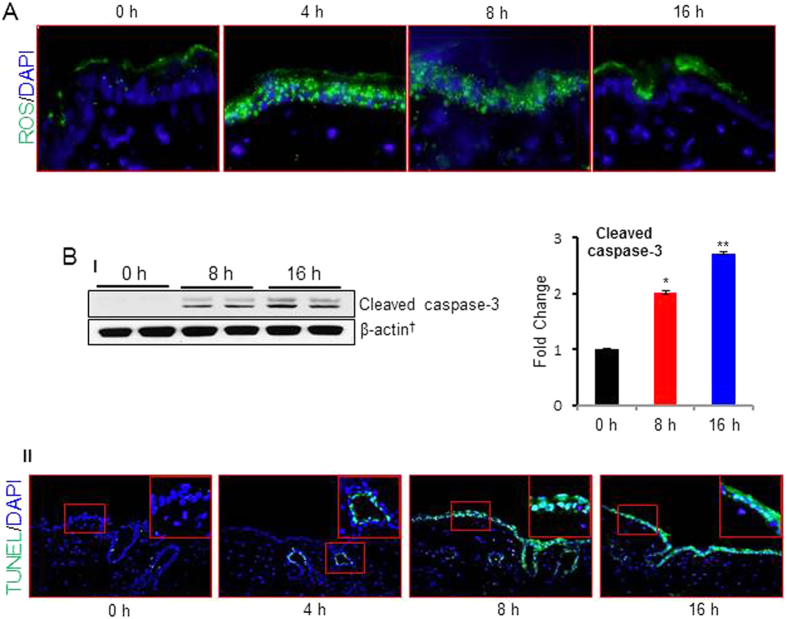
PAO induces ROS production and apoptosis in Ptch1^+/−^/SKH-1 mouse skin. Dorsal skin of Ptch1^+/−^/SKH-1 mice was treated either with vehicle or PAO (100 μg/mouse) for different time intervals (0–16 h) and assessed for ROS production and cell death markers. (**A**) Fluorescence-based microphotographs showing ROS production at various time (0–16 h) intervals as assessed by staining with CM-H2DCFDA probe. (**B**-**I**) Western blot analysis of cleaved caspase-3. Histogram showing dentiometric analysis of western blots. (**B**-**II**) Microphotographs analysis of fluorescent-based green TUNEL positive cells observed at various time (0–16 h) points. TUNEL positive cells were observed as early as within 4 h following exposure with PAO to mouse skin. Please note that apoptotic cells at this time were only found in hair follicles. However, at later time-points inter-follicular epidermis was also positive. Insets represent magnified area of the TUNEL positive cells.

Earlier studies showed that PAO is a strong oxidant^31^. To test whether PAO induces ROS production in the skin and whether cutaneous damage following its treatment is mediated by the ROS, we employed respectively, DCFH-DA and TUNEL staining of the frozen sections to assess ROS generation and apoptosis. PAO-challenged skin showed enhancement in ROS production at 4 and 8 h time-points but it was reduced at 16 h (Fig. 2A). Next, employing serial sections we assessed whether kinetics of ROS production overlaps with that of apoptosis induction. In this regard, we observed that production of ROS at early time intervals triggered apoptosis at later time-points. Both TUNEL-positive cells and cleaved caspase-3 expression were found to be consistent with this notion (Fig. 2BI, II). Interestingly, at 4 h PAO initiated the apoptosis as assessed by the TUNEL-positive hair follicle keratinocytes which at later time-points was more widespread covering both inter-follicular and follicular keratinocytes (Fig. 2B-II). Both lewisite and sulfur mustard-induced inflammatory responses and cell death in the skin keratinocytes were shown to be associated with ROS generation^36^,^38^,^39^,^40^,^41^.

### PAO-induced UPR signaling pathway regulates inflammatory responses in the skin

**Figure 3:**
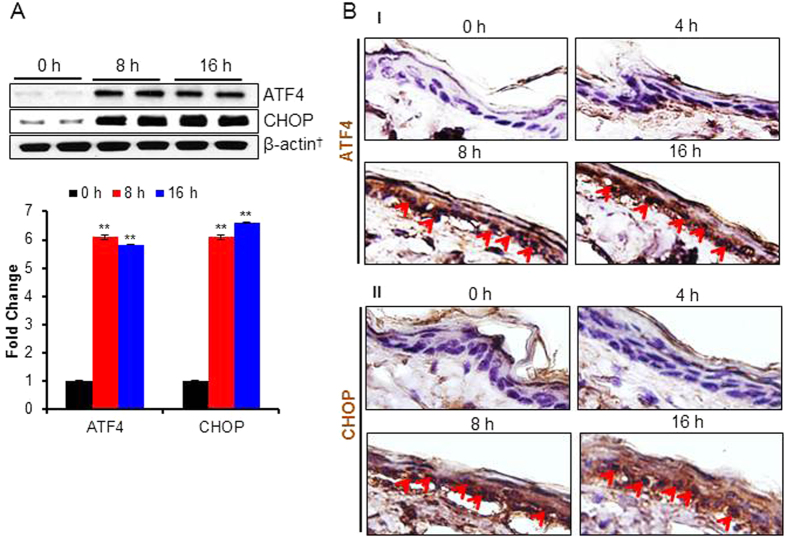
PAO-induces UPR signaling pathway in Ptch1^+/−^/SKH-1 mouse skin. In this experiment, UPR signaling pathway markers were assessed in the skin of Ptch1^+/−^/SKH-1 mice treated either with vehicle or PAO (100 μg/mouse) for different time intervals (0–16 h). (**A**) Western blot analysis of ATF4 and CHOP in the skin tissue lysate of PAO-treated samples at 0 h, 8 h and 16 h. Histogram showing dentiometric analysis of western blots. (**B**) Immunohistochemical staining of ATF4 and CHOP. Arrows showing expression and nuclear translocation of ATF4 (**B**-**I**) and CHOP (**B**-**II**) in the PAO-treated skin sections.

Earlier, we showed that arsenic treatment activates UPR signaling^42^. Here, we examined whether PAO which contains arsenic in its chemical structure also induces UPR signaling in the skin. We noted a time-dependent increase in the expression of UPR transcription factor, ATF4 and its downstream pro-apoptotic protein CHOP (Fig. 3A). In this regard, the immunohistochemical staining confirmed the results of western blot analysis in PAO-treated skin (Fig. 3B). The increase in the epidermal CHOP could be correlated with the nuclear localization of ATF4 in time-dependent manner (Fig. 3B-I,II), further confirming that CHOP is regulated by ATF4 in this experimental setting too.

**Figure 4:**
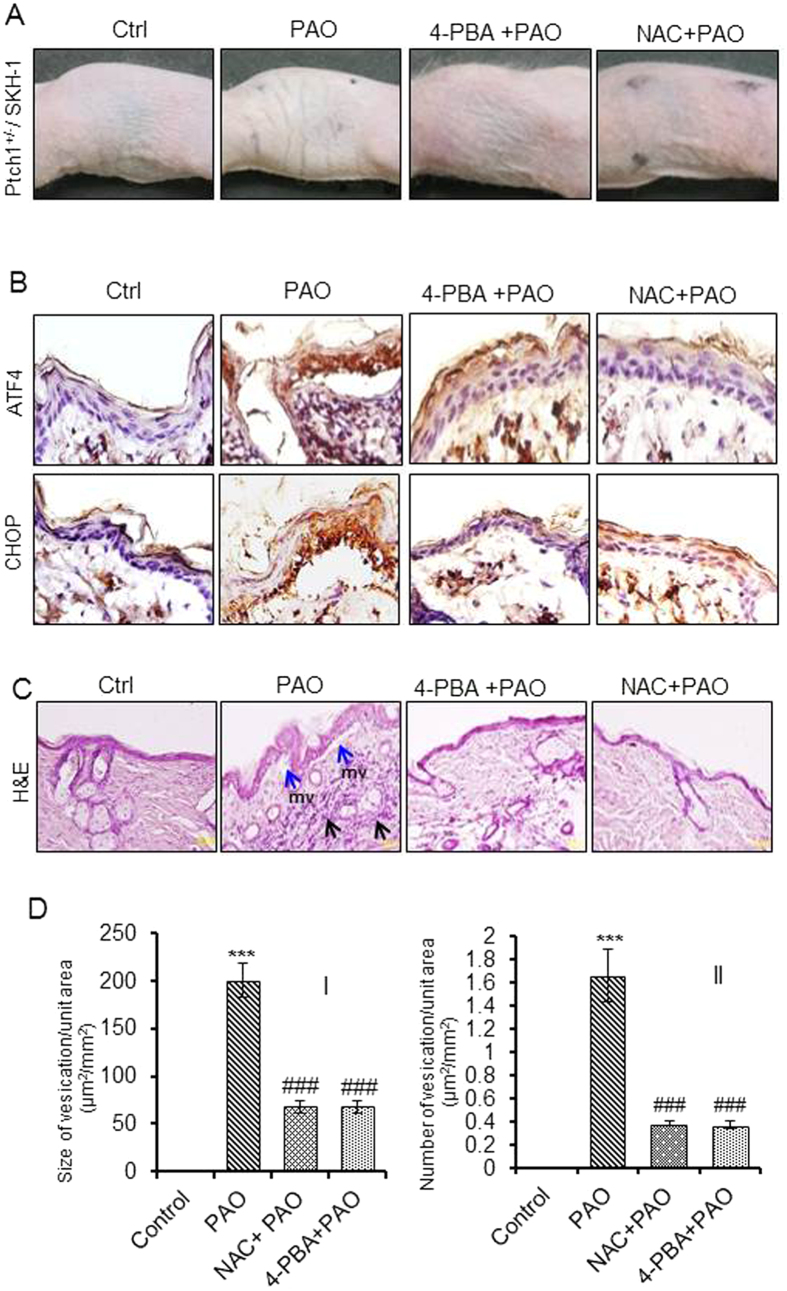
Effects of 4-PBA and NAC in PAO-induced pathological changes in Ptch1^+/−^/SKH-1 mouse skin. Mice were treated topically with either vehicle or PAO (100 μg/mouse) on the dorsal skin area followed by 4-PBA (4 mg/mouse) or NAC (5 mg/mouse) at 5 min post PAO challenge. Mice were euthanized at 8 h and skin samples were collected for histochemical analysis. (**A**) Mouse skin photographs show topical efficacy of 4-PBA and NAC on PAO-induced skin gross changes. PAO-treated mouse skin show thick, leathery, and wrinkled skin while application of 4-PBA and NAC at 5 min post PAO challenge diminishes these effects. (**B**) Immunohistochemical staining of ATF4 and CHOP. 4-PBA and NAC effectively reduces PAO-induced expression and nuclear localization of ATF4 and CHOP in epidermal keratinocytes. (**C**) Histochemical (H&E) analysis of the skin sections. Blue arrows represent mv formation and black arrows represent inflammatory leukocytes infiltrations. (**D**) Topical efficacy of 4-PBA and NAC on murine skin show effects of 4-PBA and NAC on PAO-induced size (I) and number (II) of micro-vesicants formation in murine skin. Data are expressed as mean ± SEM. ***P < 0.001 when compared to control. ^###^P < 0.001 when compared to PAO-treated group.

Then, we asked whether PAO-induced inflammatory signaling is regulated by the activation of UPR signaling pathway in the skin. For this, we used 4-phenylbutyric acid (4-PBA), a chemical chaperone which is known to reduce ER stress and inhibits ER stress induced UPR signaling^36^. 4-PBA (4 mg/mouse) applied topically on to the mouse skin 5 min post PAO challenge reduced PAO-induced UPR signaling (Fig. 4A,B). Immunohistochemical staining shows that 4-PBA also reduces nuclear localization of ATF4 and CHOP in the epidermal keratinocytes (Fig. 4B). The histochemical analysis of the skin sections revealed that 4-PBA significantly reduces both the size and number of PAO-induced micro-vesicants (Fig. 4C,D). Similarly, the inflammatory leukocytes infiltration in the dermis was also reduced following 4-PBA administration of PAO-treated mice (Fig. 4C).

### PAO-induced ROS is upstream to UPR signaling pathway and UPR-regulated inflammatory responses in the skin

Here, we tested whether blocking PAO-induced ROS by NAC treatment attenuates PAO-induced UPR signaling and UPR-regulated inflammatory responses in the skin. NAC treatment reduces UPR signaling as well as inflammatory responses including micro-blister formation (Fig. 4B–D). Consistent with the known role of ROS in upregulating UPR signaling pathway^43^, our observations confirm that ROS is upstream regulator of both UPR signaling and onset of inflammatory responses in PAO-treated skin. Furthermore, since blockade of PAO-generated ROS reduces inflammation in a UPR dependent manner, our data indicate that these three responses are tightly interlinked.

### Dose-and time-dependent effects of PAO on the production of cytokines, ROS and induction of apoptosis in human keratinocytes

**Figure 5:**
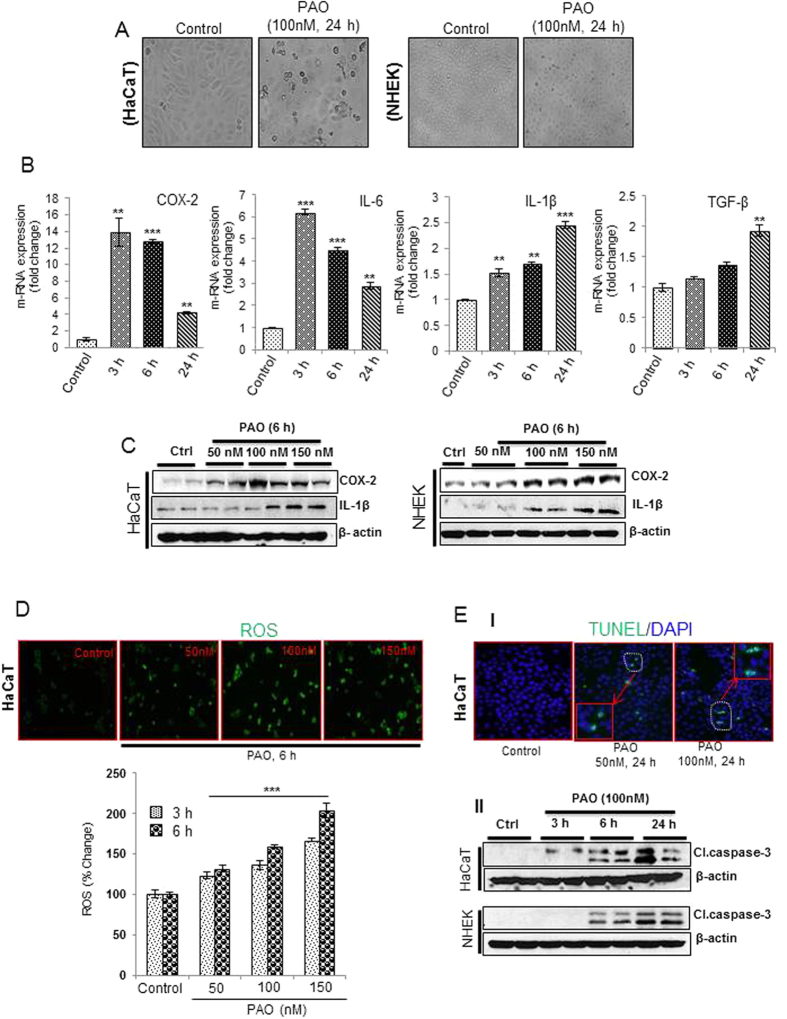
Dose- and time-dependent kinetics of cytokines production, ROS generation and apoptosis induction in PAO-treated human keratinocytes. In these experiments, human skin keratinocytes (HaCaT/NHEK) were treated with PAO (50–150 nM) for 3–24 h time intervals. (**A**) Phenotypic alterations including cell rounding, loss of cell adhesion and blebbing in HaCaT and NHEK in culture. (**B**) Real time PCR showing time-dependent response for pro-inflammatory cytokines (IL-6, IL-1β & TGF-β) and COX-2 expression. (**C**) Western blot analysis showing dose-dependent effects of PAO on the expression level of IL-1β and COX-2 in HaCaT and NHEK. (**D**) Fluorescence-based microphotographs and ELISA-based plate reader assays showing dose and time-dependent effects of PAO on ROS production in HaCaT cells. (**E**-**I**) Microphotographs of fluorescent TUNEL-positive cells at various doses of PAO. (**E**-**II**) Time-dependent effects of PAO on western blot analysis of cleaved caspase-3 in HaCaT and NHEK. Data are expressed as mean ± SEM. **P < 0.01 and ***P < 0.001 values show significance levels when compared to control.

To demonstrate whether PAO-mediated alterations in murine skin can be replicated by the human skin keratinocytes and to probe the molecular mechanism underlying PAO challenge, we employed both normal (NHEK) and immortalized (HaCaT) human epidermal keratinocytes. The selection of dose of PAO in this *in vitro* acute study is based on MTT assay (data not shown). PAO (100 nM) treatment resulted in the morphological alterations in both NHEK and HaCaT cells which could be visualized as early as 6h (data not shown). However, more pronounced wide spread and significant changes could be observed at 24 h (Fig. 5A). These phenotypic alterations include cell rounding, loss of cell adhesion and blebbing. Although, we observed similar effects in both of these keratinocytes, NHEK cells appears to be relatively more resistant than the HaCaT cells to PAO in terms of manifesting these morphological changes.

Real time PCR analysis showed that PAO induced time-dependent expression of pro-inflammatory mediators such as cyclooxygenase (COX-2) and cytokines IL-6, IL-1β and TGF-β both in HaCaT (Fig. 5B) and in NHEK cells (Supplementary Fig. S2). Results from western blot analysis also confirmed this dose-dependent response (Fig. 5C and Supplementary Fig. S3).

To confirm our *in vivo* results, we next, tested ROS production in PAO-treated keratinocytes. Fluorescence microphotographs and ELISA-based plate reader study showed that PAO induces ROS generation in dose- and time-dependent manner (Fig. 5D). We also tested whether PAO treatment leads to induction of apoptosis in these human skin keratinocytes. Indeed, PAO induced apoptosis accompanied ROS generation. A dose-dependent increase in TUNEL-positive cells (Fig. 5E-I) and expression of cleaved caspase-3 in both HaCaT and NHEK cells could be observed (Fig. 5E-II and Supplementary Fig. S4).

### PAO activates PERK/ATF4 and IRE1α/XBP-1 branch of UPR signaling in the skin keratinocytes

**Figure 6:**
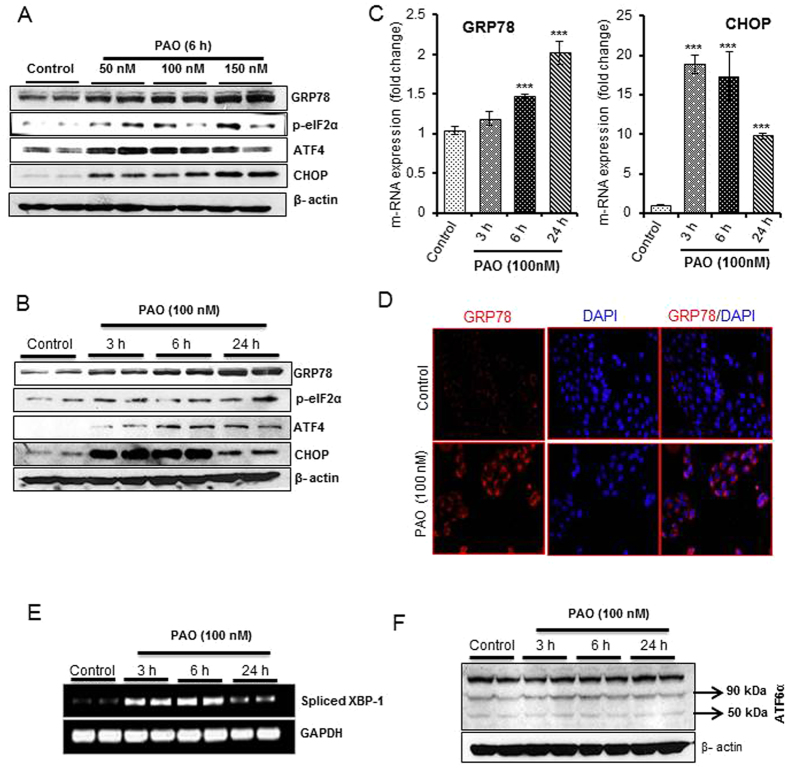
PAO activates PERK/ATF4 and XBP-1 branch of UPR signaling in human keratinocytes. (**A**,**B**) Western blot analysis showing dose- and time -dependent increase in UPR signaling pathway proteins GRP78, p-eIF2α, ATF4 and CHOP following PAO treatment in HaCaT cells. (**C**) Real time PCR analysis of PAO-induced GRP78 and CHOP mRNA expression in HaCaT cells. (**D**) Immunofluorescence staining of PAO-induced GRP78 in HaCaT cells. (**E**) Semi-quantitative PCR analysis of spliced XBP-1 (sXBP-1) in PAO-treated and vehicle-treated HaCaT cells. (**F**) Western blot analysis of spliced ATF6α (50 kDa) in PAO-treated and vehicle-treated HaCaT cells. Data are expressed as mean ± SEM. ***P < 0.001 show significance levels.

Next, we examined the effects of PAO on all the three known UPR signaling pathway sensor proteins PERK, IRE1α, and ATF6α^44^. Dose- and time-dependent activation of PERK-ATF4-dependent UPR signaling pathway regulatory proteins GRP78, p-eIF2α, ATF4 and CHOP have been found to occur in HaCaT cells (Fig. 6A,B and Supplementary Fig. S5). Real time PCR analysis (Fig. 6C) and immunofluorescence staining of UPR chaperone GRP78 revealed similar results (Fig. 6D). PAO-treated HaCaT cells also showed high expression of spliced XBP-1 (sXBP-1) (Fig. 6E), while significant changes in spliced ATF6α (spliced 50 kDa) could not be observed (Fig. 6F and Supplementary Fig. S6). These results suggest that PAO induces UPR signaling pathway in keratinocytes mainly via PERK/ATF4 and IRE1α/XBP-1 branches. The effects of PAO in NHEK were more or less similar to those observed in HaCaT cells (Supplementary Fig. S7).

### CHOP plays a central role in PAO-induced apoptosis and cytokines production

**Figure 7:**
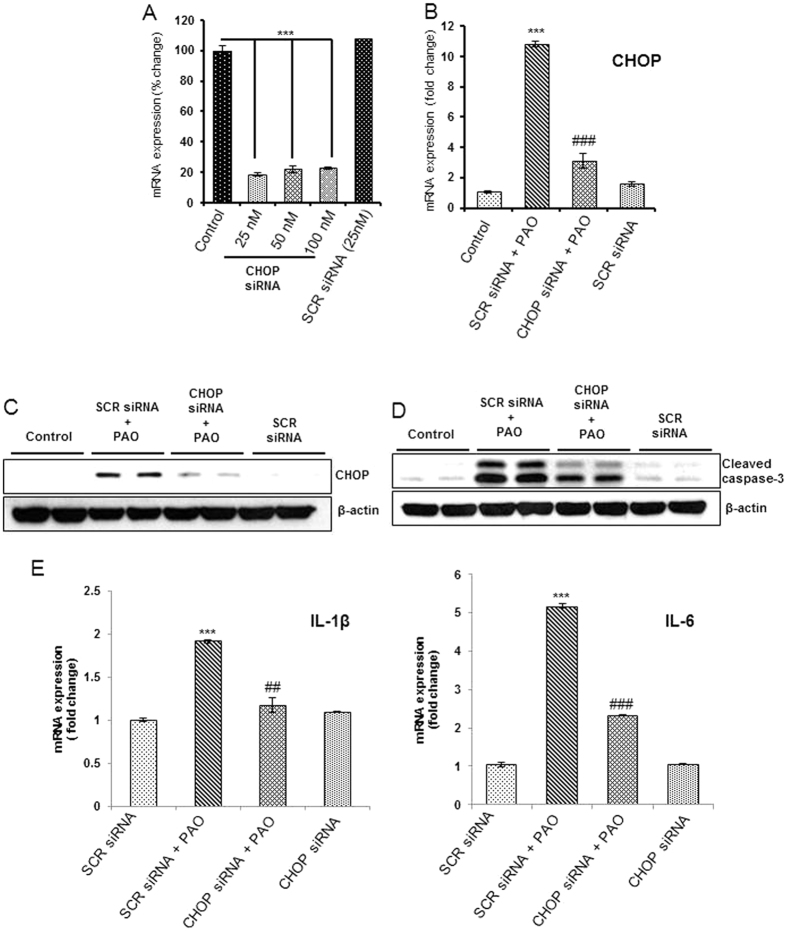
Ablation of CHOP expression attenuates cytokines expression and apoptosis in PAO-treated human keratinocytes. Effects of PAO (100 nM, 24 h) on cytokine expression and apoptosis were examined in HaCaT cells transfected with either CHOP siRNA or scrambled (SCR) siRNA. (**A**) Knockdown effects of CHOP siRNA (25–100 nM) observed by real time PCR analysis. (**B**,**C**) Real time PCR and western blot expression analysis showing effects of CHOP siRNA (25 nM) on PAO-induced CHOP expression. (**D**) Western blot expression analysis of cleaved caspase-3 in cell lysate obtained from HaCaT cells transfected with either SCR siRNA or with CHOP siRNA in the presence of PAO. (**E**) Real time mRNA expression analysis of IL-1β and IL-6 in the cells transfected with either SCR siRNA or with CHOP siRNA in the presence of PAO (100 nM, 6 h). Data are expressed as mean ± SEM. ***P < 0.001 when compared to control. ^##^P < 0.01, ^###^P < 0.01 when compared to PAO-treated group.

CHOP is one of the key UPR regulatory cytotoxic protein^45^. Since, we observed that PAO induced CHOP in the skin keratinocytes both *in vivo* and *in vitro*, we further explored its role in PAO-induced apoptosis and inflammatory signaling using RNA interference-based approaches. In keratinocytes where CHOP expression following CHOP siRNA transfection was reduced by about 80% (Fig. 7A) as compared to scrambled siRNA transfected negative control keratinocytes, we confirmed reduced CHOP expression following PAO treatment as ascertained by both real time PCR and western blot data (Fig. 7B,C and Supplementary Fig. S8). Consistently, PAO-induced cleavage of caspase-3 (Fig. 7D and Supplementary Fig. S8) and augmented pro-inflammatory cytokine IL-1β and IL-6 production were diminished in these CHOP-ablated cells (Fig. 7E). These data suggest a role of CHOP in PAO-induced cytokine production and cell death. Interestingly, sXBP-1 was also found to be reduced in CHOP siRNA transfected cells (Supplementary Fig. S9A). However, we could not detect significant changes in PAO-induced COX-2 expression in CHOP-ablated cells (Supplementary Fig. S9B), suggesting that PAO-mediated regulation of COX-2 occurs via alternate mechanism.

### 4-phenylbutyric acid and N-acetylcysteine attenuate PAO-induced ROS production, UPR/inflammatory signaling and apoptosis in skin keratinocytes

**Figure 8:**
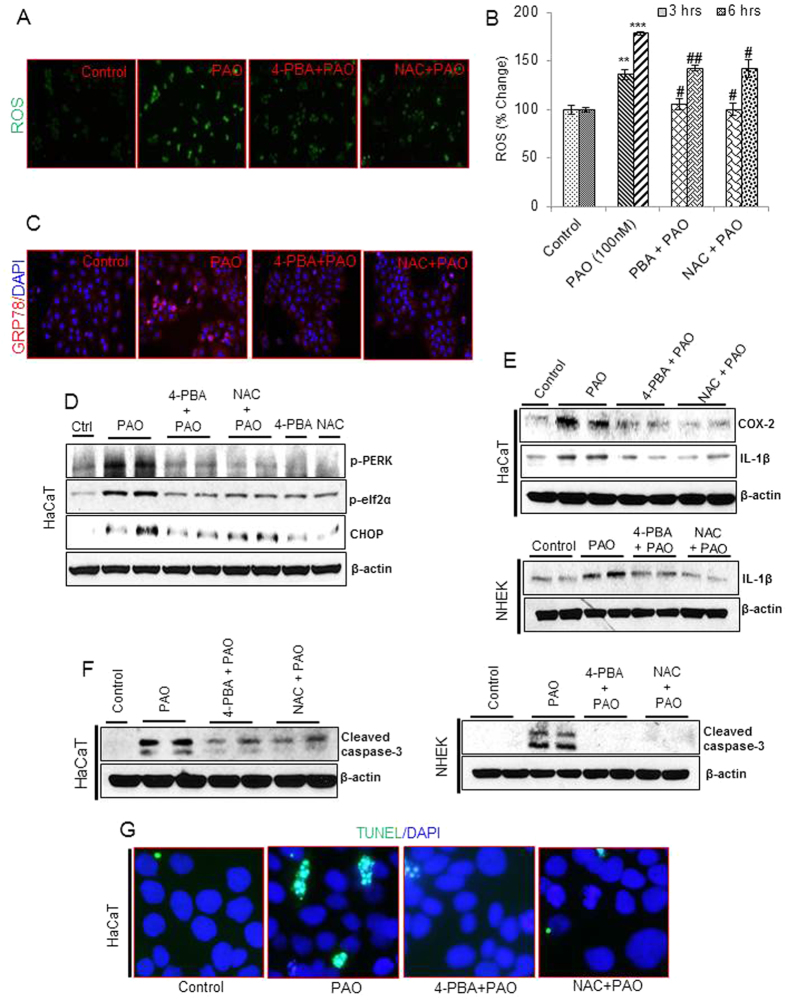
Co-treatment with 4-PBA or NAC attenuates PAO-induced changes in ROS, UPR signaling proteins, inflammatory cytokines and apoptosis in human skin keratinocytes. UPR, inflammatory and apoptosis regulatory markers were examined in PAO (100 nM, 24 h)-treated HaCaT and NHEK cells in the presence or absence of 4-PBA (1 mM) or NAC (10 mM) as a co-treatment. (A & B) Fluorescence-based microscopic pictures and ELISA-based plate reader data analysis of ROS (% change) in HaCaT cells. (**C**) Immunofluorescence staining of GRP78 in HaCaT cells. (**D**) Western blot expression analysis of p-PERK, p-eIF2α and CHOP in HaCaT cells. (**E**) Western blot expression analysis of COX-2 and IL-1β in HaCaT and NHEK lysate. (**F**) Western blot expression analysis of cleaved caspase-3 in HaCaT and NHEK lysate. (**G**) Microscopic picture analysis of green fluorescent TUNEL positive HaCaT cells. Data are expressed as mean ± SEM. **P < 0.01, ***P < 0.001 when compared to control. ^#^P < 0.05, ^##^P < 0.01 when compared to PAO-treated group.

Next, we explored the role of PAO-induced ROS-mediated UPR signaling in regulating inflammatory response and apoptosis in these skin keratinocytes. For this, we employed both antioxidant, NAC and chemical chaperone, 4-PBA. Fluorescence microphotographs (Fig. 8A) and ELISA**-based plate assays (Fig. 8B) showed that 4-PBA and NAC both significantly reduced PAO-induced ROS generation in HaCaT cells. These treatments significantly reduced PAO-induced expression of GRP78 (Fig. 8C). Western blot data analysis of cell lysates revealed that both 4-PBA and NAC treatment could lower the expression of p-PERK, p-eIF2α and CHOP in PAO-treated HaCaT (Fig. 8D and Supplementary Fig. S10) and NHEK cells (Supplementary Fig. S11). These results were confirmed by both real time PCR and immunofluorescence staining analysis (Supplementary Fig. S12). Similarly, these agents abrogated PAO-induced inflammatory cytokines production and apoptosis in these skin keratinocytes. Consistently, PAO-induced mRNA expression of COX-2, IL-1β and IL-6 were diminished almost to control levels by both 4-PBA and NAC treatments (Supplementary Fig. S13). Western blot analysis confirmed the results of real time PCR at protein level in both of these keratinocytes (Fig. 8E and Supplementary Fig. S14). Similarly, both of these agents also diminished PAO-induced cleaved caspase-3 in HaCaT and NHEK cells (Fig. 8F and Supplementary Fig. S15) as well as number of TUNEL-positive cells in PAO-treated keratinocytes (Fig. 8G). The comparable efficiency of both NAC and 4-PBA in attenuating PAO-mediated responses indicate a tight link between induction of ROS and UPR signaling and inflammatory and apoptosis regulatory process.

## Discussion

Chemical warfare agents (CWAs) such as arsenicals are chemical substances that can be used to destroy, injure or incapacitate enemy in warfare. Although with the implementation of various war accords particularly with the efforts of the Organization for the Prohibition of Chemical Weapons (OPCW)^36^,^46^, large stockpiles of these chemicals are destroyed. However, still some of these agents are known to exist. Thus, CWAs could be potential threat against civilian populations in terrorist attack or due to accidental exposure. Taking these concerns into account there is a need to develop mechanism-based effective antidotes and treatments against these agents. The toxicity and vesicant inducing activity of lewisite in comparison to other similar CWAs is considered to be much higher in the magnitude and much faster as well^8^,^9^. However, due to technical difficulties in using these agents in laboratory setting, there is a need to define a less toxic surrogate arsenical with which the pathogenesis of these agents could be studied.

Our data as described here show that PAO, a lewisite analog, and also a degradation product of CWAs causes damage to murine skin which is similar to that reported for lewisite^20^,^34^,^36^. Thus, these skin lesions which include microvesications, blisters and severe inflammation are associated with ROS generation, activation of UPR signaling pathway and apoptosis could be observed by exposure to both PAO and lewisite^36^. Our data showing that blockade of ROS generation by administrating antioxidant NAC diminishes both UPR signaling and inflammation/blistering suggest that PAO-induced ROS is involved in the regulation of UPR signaling and associated tissue injury. In this regard, it is known that ROS regulates UPR signaling pathway under various experimental settings including following lewisite exposure^36^,^43^,^47^. Similarly, administration of chemical chaperone, 4-PBA diminished tissue lesions including massive keratinocytes cell death suggesting that ROS regulated UPR signaling orchestrates both cutaneous inflammation and tissue damage. Earlier studies also described a role of UPR signaling in regulating inflammatory response in various other diseases models^48^,^49^. In addition, we also showed that inorganic arsenic such as sodium arsenite and arsenic trioxide under multiple subacute exposure setting causes UPR signaling induction associated with mild inflammatory responses in the skin^42^,^47^. Thus, it appears that arsenic of PAO which may be released following its decomposition *in situ* could contribute to the PAO-mediated UPR signaling and cutaneous inflammation. However, since the severity and rapidity of these responses caused by PAO are several folds higher than that caused by inorganic arsenic, it is likely that the organic moieties associated with arsenic could contribute to these differences particularly due to much enhanced tissue penetration ability of PAO and other similar structurally related arsenicals. In addition, since the manifestations of these structurally different arsenicals may also differ to some degree, it is likely that some molecular targets of these arsenicals in various tissues may be distinct. However, as in the skin many of these responses as observed in case of PAO and lewisite are not much different, it could be speculated that following penetration in the skin these chemicals invoke initial identical molecular trigger.

The observed differences between *in vivo* and *in vitro* in the expression of inflammatory mediators such as cytokines may be due to generation of these mediators by multiple cell types *in vivo*. Interestingly, our observations that chemical chaperone treatment diminishes ROS production and NAC was found to be more effective than 4-PBA suggest a close regulatory loop between the PAO-mediated ROS generation and UPR signaling. However, based on these observations we also believe that PAO-mediated induction of ROS in the skin keratinocytes could be both independent and dependent of UPR signaling pathway.

4-PBA and NAC employed in this study as well as against lewisite^36^ are FDA approved for the treatment of urea cycle disorder and acetaminophen overdose toxicity respectively^50^,^51^. We therefore believe that repurposing these agents as antidotes for arsenicals toxicity will be a significant progress in this area of research. Although based on these results we propose that PAO is a surrogate chemical for studying arsenicals toxicity, it is also important as its own as this is a degradation product of CWAs and may be highly damaging not only to experimental animals but also to humans. For example DPAA and PAO besides some other arsenic compounds were found to be present in drinking water in Kamisu, Japan and exposed population consuming contaminated water supply developed a toxic syndrome with brain stem-cerebellar and cerebral symptoms^14^. However, skin conditions in this population was not reported. These contaminants were considered to be present due to the degradation of diphenylchlorarsine and diphenylcynoarsine^14^. Similarly, at a road construction site in Samukawa, Kanagawa where the Sagami Naval Arsenal was formally located, several hundred beer bottles were unearthed of which more than 200 contained lewisite^16^. Although greater details of this accident remained unknown but laborers working there were described to be injured when first group of bottles were discovered^16^.

In summary, we describe here PAO as a surrogate chemical which could be effectively used to define the molecular pathogenesis associated with the exposure to arsenicals particularly in the skin. This agent could also be used to define the novel therapeutic agents that could block the acute and chronic toxic manifestations of arsenicals and other related degradation products. Additional screening studies using PAO could be performed in *in vitro* high-throughput setting as well as in *in vivo* setting to develop drugs for therapeutic intervention of diseases/disorders or conditions where unfolded/misfolded proteins are known to be involved in their pathogenesis.

## Methods

### Cell lines and reagents

Immortalized human skin keratinocytes (HaCaT) and normal human epidermal skin keratinocytes (NHEK) were obtained from AddexBio Technologies (San Diego, CA) and Lonza (Basel, Switzerland) respectively. HaCaT cells were maintained in DMEM medium (Hyclone, (South Logan, UT) containing 10% fetal bovine serum (Sigma, St. Louis, MO), 1% penicillin streptomycin solution (Mediatech, Manassas, VA) at 37 °C in 5% CO_2_ incubator, while NHEK were cultured in KBM-Gold keratinocyte cell basal medium (Basel, Switzerland) supplemented with KGM-Gold SingleQuotes (Basel, Switzerland). N-acetylcysteine (NAC), 4-Phenylbutyric acid (4-PBA) and phenylarsine oxide (PAO) were obtained from Sigma (St. Louis, MO). 2′,7′- dichlorodihydrofluorescein diacetate (CM-H2DCFDA) and DAPI were obtain from Life Technology (Carlsbad, CA). CHOP (DDIT3) siRNA (Cat. no. SI00059528) was obtained from Qiagen (Hilden, Germany). Real Time PCR primers used in the study were obtained from Invitrogen (Carlsbad, CA) and listed in Supplementary Table S2.

### PAO-preparation and treatment

PAO was dissolved in 100% ethanol in a continuously operated chemical and biological hood and treatment was done under the safety laminar hood using all required approvals using personel protective equipment. 3M™ 6000 series reusable half face piece respirator (Fisher, Pittsburgh, PA) equipped with 3M 6003 organic vapor/acid gas cartridge was used during PAO preparation and mice exposure. PAO challenged animals were kept in continuously operated chemical and biological hood and were observed throughout the exposure period before killing.

### Cell culture studies

A 1 M stock solution of PAO was prepared fresh in 100% ethanol by warming at 37 °C for 5–10 min. Cells were treated either with vehicle control or with various concentration of PAO (50–150 nM) diluted in culture medium and studies were carried out between time-points 3 to 24 h. For assessing protection, NAC (10 mM) or 4-PBA (1 mM) was used as co-treatment to PAO-treated cells. Cells were treated each time when confluency reached to about 70–80%.

### Animal studies

Prior to PAO application on the mice skin, pain was managed by subcutaneous injection of buprenorphine at a dose of 0.05–0.1 mg/kg approximately 30 min prior to anesthesia. Mice were anesthetized with ketamine and xylazine (100 mg/kg for ketamine and 5 to 7 mg/kg for xylazine) by intraperitoneal (I.P.) injection. Once the mice were anesthetized, an exposure area (1.6 × 1.6 cm^2^) was marked by indelible marker on the dorsal surface of the mice skin, and then the mice were separated into two groups (n = 5, each group). Control mice group was applied with vehicle (ethanol, 30 μL). Whereas experimental group of mice was treated with 30 μL diluted PAO (100 μg/mouse) topically on the marked dorsal skin of mice. Dose selection of PAO in this *in vivo* study was based on earlier published report^28^. PAO-induced appearance and progression of lesions (e.g. erythema, edema, wounding, tissue damage etc.) on the skin were photographed and evaluated in terms of draize scores as detailed in Supplementary Table S1. Following PAO application skin bi-fold thickness in millimeter (mm) was measured at 0, 4, 8 and 16 h time-points with the help of an electronic digital caliper. Mice were euthanized at 0, 4, 8 and 16 h and skin samples were collected and immediately freeze in liquid nitrogen for further analysis. 4-PBA (4 mg/mouse) and NAC (5 mg/mouse) was applied topically on to the mouse skin at 5 min post PAO challenge for efficacy studies. Since 4-PBA has lower solubility in water, thus a universal vehicle (a mixture of 1/3 H_2_O, 1/3 ethanol, and 1/3 glycerol) was formulated to easily dissolve 4-PBA and NAC^36^. All studies were conducted in accordance with protocols approved by the Institutional Animal Care and Use Committee (IACUC) of the University of Alabama at Birmingham, USA.

### Hematoxylin and eosin (H&E) staining

H&E staining in the skin sections was performed as described earlier^52^. Skin tissues were fixed in 10% formalin, embedded in paraffin and were sectioned (5 μm) on to glass slides using microtome (Thermo scientific, Grand Island, NY). These skin sections were deparaffinized in xylene, rehydrated and stained with H&E.

### Immunofluorescence (IF) staining

For immunofluorescence staining, skin sections were deparaffinized, rehydrated and then incubated in antigen unmasking solution according to the manufacturer’s instructions (Vector laboratories, Burlingame, CA). A blocking buffer of 2% bovine serum albumin in PBS for 30 min at 37 °C was used to avoid non-specific binding of antibodies and then incubated with primary antibodies. The sections were then incubated with fluorescence-coupled secondary antibody and visualized under fluorescence microscope. Immunofluorescence staining of keratinocytes cells was performed in 4% paraformaldehyde fixed and 0.1% Triton X-100 permeabilized cells. Antibodies labeled cells were examined using fluorescence microscopy.

### Immunohistochemistry (IHC)

IHC analysis of the skin sections were carried out as described earlier^53^. In brief, 5 μM thin skin sections were de-paraffinized following rehydration and then incubated in antigen unmasking solution according to the manufacturer’s instructions (Vector laboratories, Burlingame, CA, USA). To avoid nonspecific binding of antibodies, a blocking buffer of 2% BSA in PBS for 30 min at 37 °C was used and then these sections were incubated with primary antibodies followed by a universal peroxidase-coupled secondary antibody and visualized with DAB substrate.

### Morphological changes

Morphological changes were assessed at 24 h following treatment with vehicle or PAO (100 nM) to HaCaT and NHEK. Keratinocytes were observed for phenotypic alterations (cell roundness, cell blebbing, and detachment etc.) from the normal polyhedral morphology under the phase contrast microscopy (Olympus1X-S8F2, Japan).

### Reactive Oxygen Species (ROS)

ROS generation was assessed using fluorescent probe diclorodihydrofluorescein di-acetate (CM-H2DCFDA) as described earlier^42^. CM-H2DCFDA is a non-fluorescent dye but switched to highly fluorescent dichlorofluorescein (DCF) when oxidized by intracellular ROS. In brief, HaCaT (1 × 10^4^ cells per well) cells were seeded in 96-well black bottom culture plates and allowed to adhere for 24 h in CO_2_ incubator at 37 °C. Following PAO treatment, cells were incubated at a final concentration of 5 μM of CM-H2DCFDA in serum-free medium for 20 min at 37 °C. The reaction mixture was then aspirated, washed and replaced by 200 μl of PBS in each well. Fluorescence intensity was measured on excitation wavelength at 485 nm and emission wavelength at 528 nm. Vehicle-treated sets were also run under identical conditions and served as control. In addition, intracellular fluorescence imaging using upright fluorescence microscope for ROS generation was also performed in parallel experiments.

To test ROS production in PAO-treated skin samples, freshly cut OCT-embedded skin cryo-sections (5 μm) were incubated with 10 μM CM-H2DCFDA probe dissolved in ACAS buffer (127 mM NaCl, 0.8 mM MgCl_2_, 3.8 mM KCl, 1.2 mM KH_2_PO_4_, 1.2 mM CaCl_2_, 5 mM glucose & 10 mM HEPES PH-7.4) for 1 h at room temperature. Slides were washed three times in dark and were mounted with mounting medium containing DAPI and visualized under fluorescent microscope.

### Quantitative real time PCR (qRT-PCR)

Quantitative real time PCR (qRT-PCR) analysis was carried out using either SsoFast Evagreen Super mix (Bio-Rad, Hercules, CA) fluorescent dye or TaqMan (Applied Biosystem, Foster City, CA) one step PCR master mix as described earlier^36^. Total cDNA (250 ng) was used in a 10 μl reaction mixture with sequence specific primers. qPCR reactions were carried out using 7500 fast Real-Time PCR system (Applied Biosystem, Foster City, CA). Cycling conditions were 20 s at 95 °C followed by 40 cycles at 95 °C for 3s and 60 °C for 30 s. Relative quantification of the steady state target mRNA levels was calculated after normalization of total amount of cDNA to GAPDH endogenous reference.

### Terminal deoxynucleotidyl transferase dUTP nick end labeling assay

TUNEL assay was performed using the *in situ* DNA fragmentation assay Kit (Roche Diagnostics, Indianapolis, IN) according to the manufacturer’s instructions. Briefly, HaCaT cells (5 × 10^4^) were grown on glass coverslips. Following PAO treatment, cells were washed with PBS, fixed with methanol and incubated with proteinase K for 15 min at 37 °C. Cells were then washed three times and incubated with 50 μM TUNEL reaction mixture for 1h at 37 °C in a humid chamber, and visualized using fluorescence microscopy.

### RNA isolation and reverse transcriptase PCR

Total RNA was extracted from cultured cells using TRIzol (Invitrogen, Grand Island, NY). m-RNA (1 μg) was reverse-transcribed into cDNA by iScript cDNA synthesis Kit (Bio-Rad, Hercules, CA). PCR products for spliced XBP-1 were run on 1.5% agarose gels and photographed using Gel Documentation System (Bio-Rad laboratories, Hercules, CA). GAPDH was used as endogenous control.

### Protein quantification and western blot analysis

Protein lysates were prepared using an ice cold lysis buffer (Bio-Rad, Hercules, CA) following protein assay using a DC kit (Bio-Rad, Hercules, CA). These lysates were mixed with 5X sample buffer, boiled for 5 min at 95 °C and subjected to SDS-PAGE. Proteins were electrophoretically transferred to polyvinylidene difluoride membrane and then nonspecific site were blocked with 5% nonfat dry milk in Tris buffer saline tween-20 (TBST) for 1 h at room temperature followed by probing with primary antibodies overnight at 4 °C. After washing, the membranes were incubated for 1.5 h with HRP conjugated secondary antibodies. The blots were developed with enhanced chemiluminescence (ECL) according to manufacturer’s instructions (Santa Cruz Biotechnology, Dallas, TX). Identical β-actin loading controls represent stripping and reprobing with the same blot as denoted by symbol (†) in various figures. Band densities were measured using Image J software and results were normalized to their corresponding β-actin. List of primary antibodies used in this study are listed in Supplementary Table S3.

### siRNA transfection

For siRNA inhibition studies, the cells were transfected with siRNA against human CHOP or scrambled siRNA (negative control) at final concentration 25 nM using lipofectamine 2000 according to manufacturer’s instruction.

### Statistical analysis

Data are presented as mean ± standard error of mean (SEM). Statistical analysis was performed using either unpaired Student’s t-test or one-way analysis of variance (ANOVA) following Bonferroni’s post hoc test for comparison between groups. *P < 0.05, **P < 0.01 and ***P < 0.001 were considered to be statistically significant when compared to control. ^#^P < 0.05, ^##^P < 0.01 and ^###^P < 0.001 were considered to be statistically significant when compared to PAO-treated group.

## Additional Information

**How to cite this article**: Srivastava, R. K. *et al*. Defining cutaneous molecular pathobiology of arsenicals using phenylarsine oxide as a prototype. *Sci. Rep*. **6**, 34865; doi: 10.1038/srep34865 (2016).

## Supplementary Material

Supplementary Information
