# Supplementary material for: Defining cutaneous molecular pathobiology of arsenicals using phenylarsine oxide as a prototype
Source: Sci Rep. 2016 Oct 11;6:34865. doi: 10.1038/srep34865 (PMC5057142; doi:10.1038/srep34865)
Supplement: Supplementary Information [file srep34865-s1.pdf]

Supplementary figures and tables file

Defining cutaneous molecular pathobiology of arsenicals using phenylarsine oxide as a prototype

Ritesh K Srivastava[1]*, Changzhao Li[1]*, Zhiping Weng[1], Anupam Agarwal[2], Craig A. Elmets[1] Farrukh Afaq[1], Mohammad Athar[1]#

[a]Department of Dermatology and Skin Diseases Research Center, University of Alabama at Birmingham, Alabama, USA.

[b]Division of Nephrology, Department of Medicine, School of Medicine, University of Alabama at Birmingham, Alabama, USA.

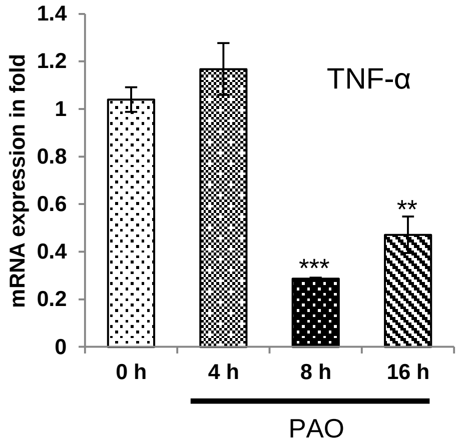

Supplementary Fig. S1: Real time PCR analysis of TNF-α in the skin of mouse either treated with vehicle or PAO (100 μg/mouse). Data are expressed as Mean ± SEM. **P<0.01 and ***P<0.001 indicate significant change when compared to control (0 h).

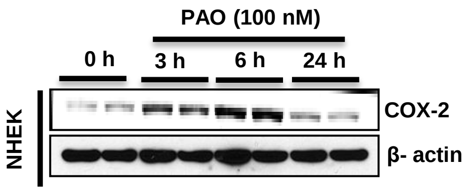

Supplementary Fig. S2: Time-dependent western blot analysis of COX-2 in the cell lysate of either PBS-treated or PAO-treated NHEK. β-actin was used as a loading control.

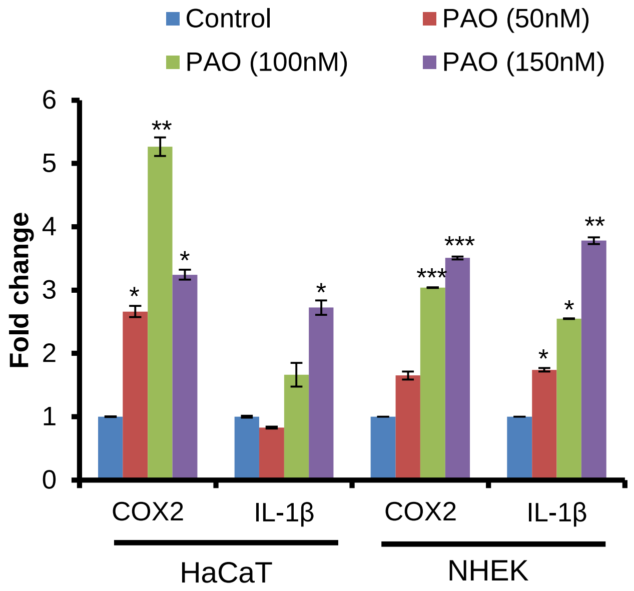

Supplementary Fig. S3: Dentiometric analysis of western blots for fig. 5C. β-actin was used as a loading control. *p<0.05, **P<0.01, ***P<0.001 when compared to their respective controls.

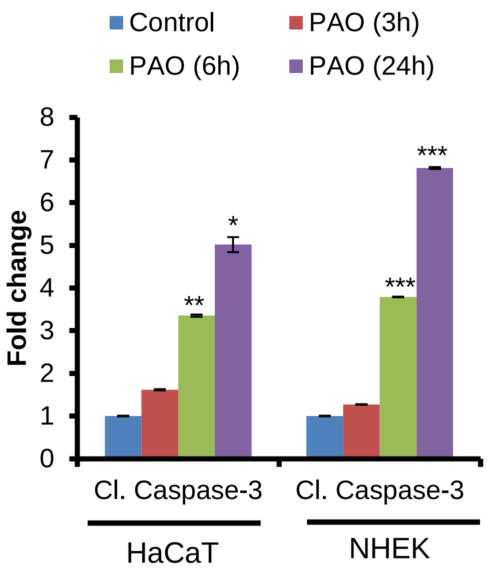

Supplementary Fig. S4: Dentiometric analysis of western blots for fig. 5E-II. β-actin was used as a loading control. *p<0.05, **P<0.01, ***P<0.001 when compared to their respective controls.

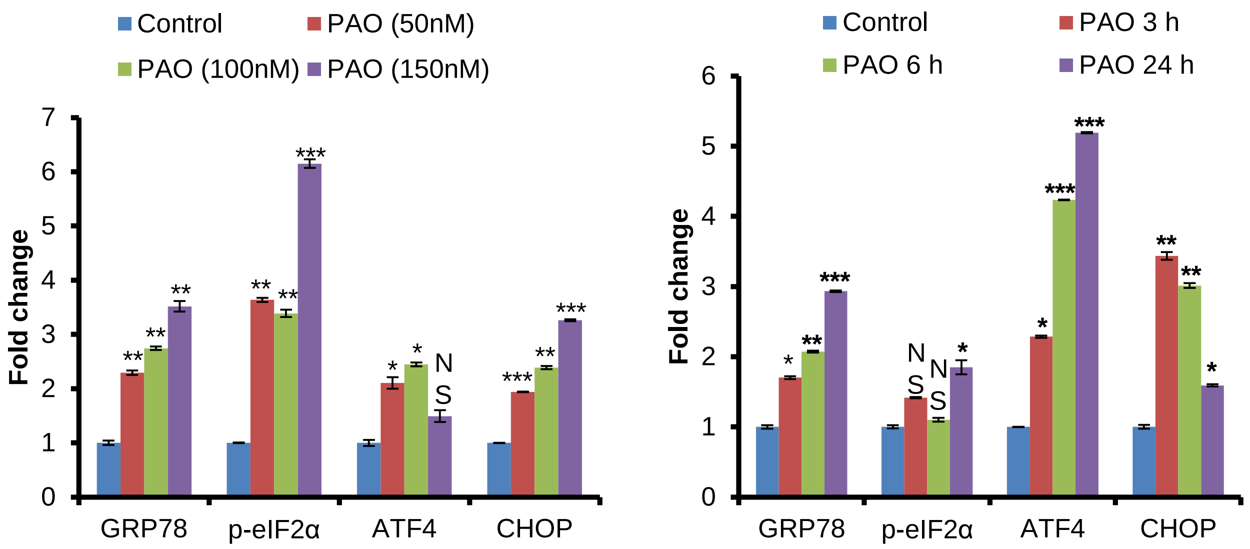

Supplementary Fig. S5: Dentiometric analysis of western blots for fig. 6A & 6B. β-actin was used as a loading control. *p<0.05, **P<0.01, ***P<0.001 when compared to their respective controls. NS- Non significant

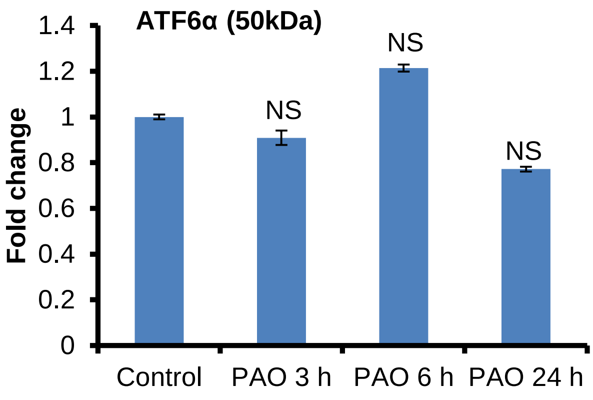

Supplementary Fig. S6: Dentiometric analysis of western blots for fig. 6F. β-actin was used as a loading control. NS- Non significant.

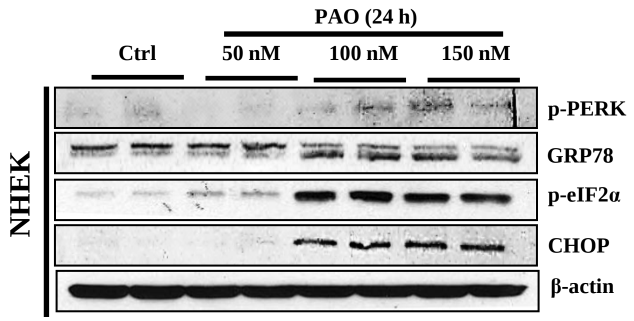

Supplementary Fig. S7: Dose-dependent western blot analysis of p-PERK, GRP78, p-eIF2α and CHOP in the cell lysate of either PBS-treated (control) or PAO-treated (50-150 nM, 24 h) NHEK. β-actin was used as a loading control.

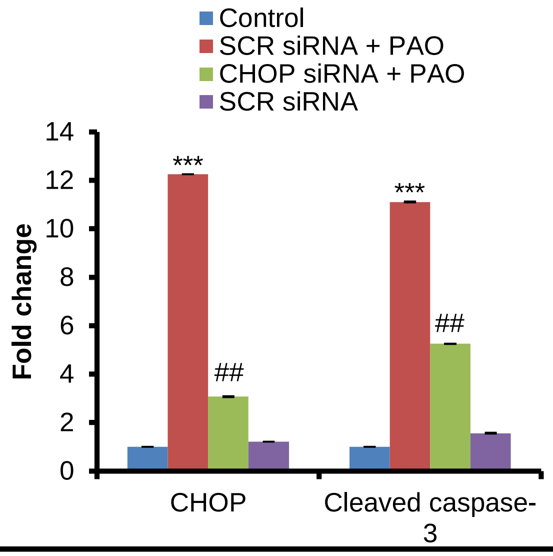

Supplementary Fig. S8: Dentiometric analysis of western blots for fig. 7C & 7D. β-actin was used as a loading control. ***P<0.001 when compared to control. $^{\#\#}$P<0.01 when compared to SCR siRNA + PAO-treated group.

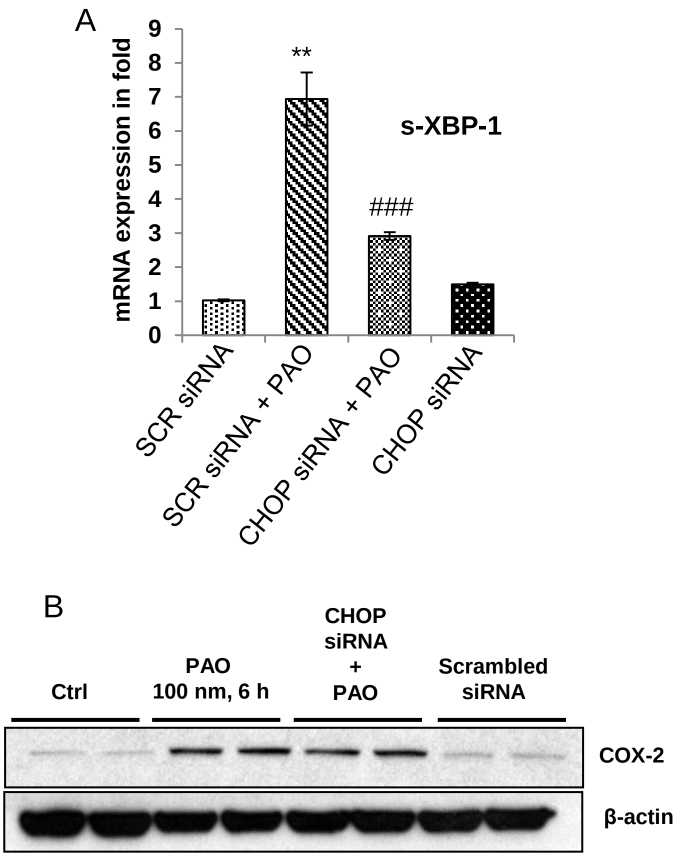

Supplementary Fig. S9: (A) Real time PCR analysis of sXBP-1 in CHOP knockdown HaCaT cells treated either with vehicle or PAO. (B) Western blot analysis of COX-2 in CHOP knockdown HaCaT cells treated either with vehicle or PAO. Scrambled siRNA was used as a negative control.

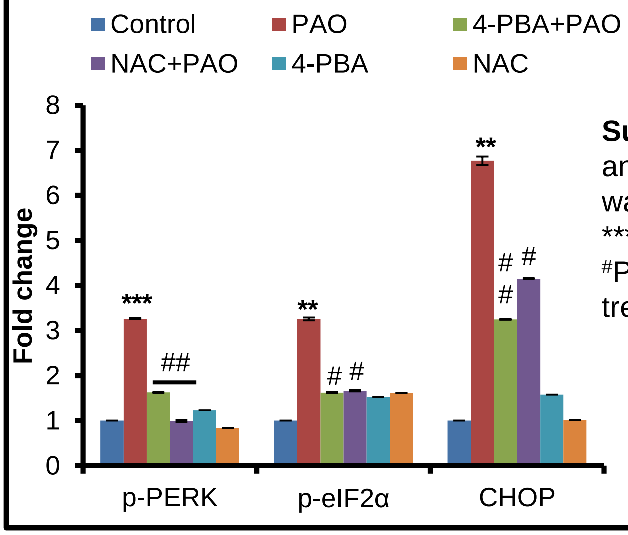

Supplementary Fig. S10: Dentiometric analysis of western blots for fig. 8D. β-actin was used as a loading control. **P<0.01, ***P<0.001 when compared to control. $^{\#}$P<0.05, $^{\#\#}$P<0.01 when compared to PAO-treated group.

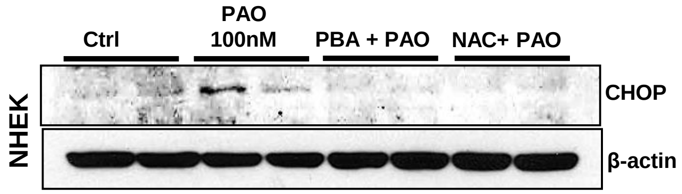

Supplementary Fig. S11: Western blot analysis of CHOP in NHEK cells treated with PAO in the presence or absence of 4-PBA or NAC.

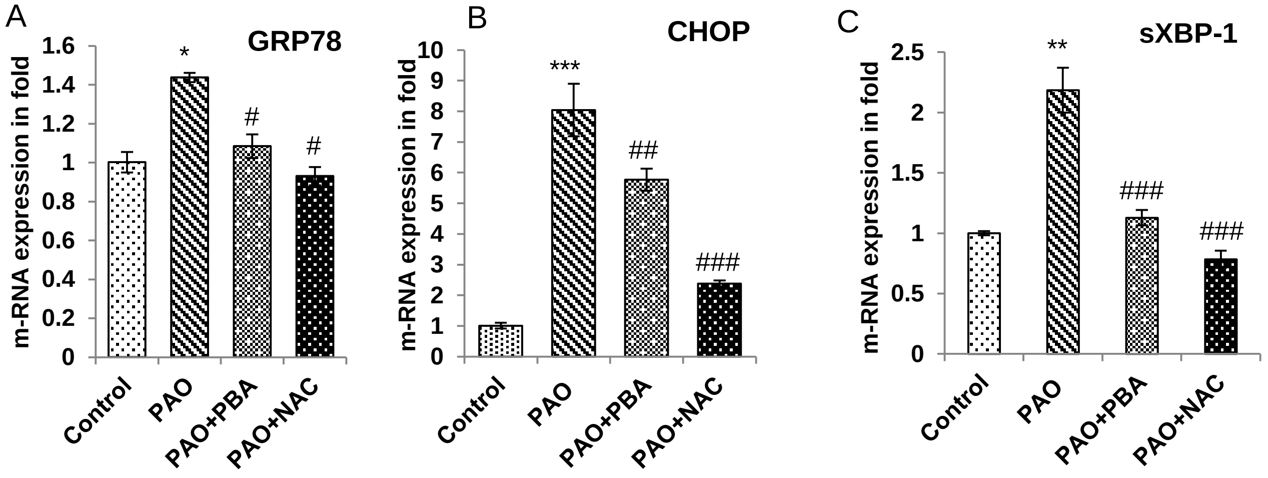

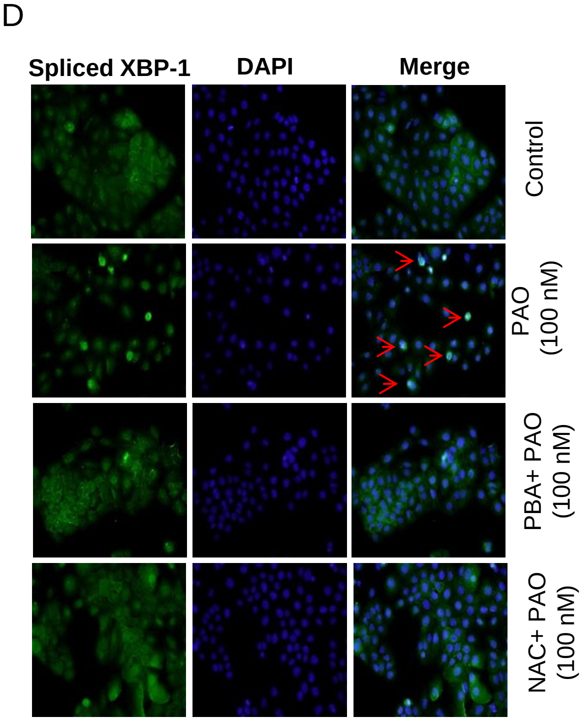

Supplementary Fig. S12: Real time PCR analysis of GRP78 (A), CHOP (B) and spliced (s) XBP-1 (C) in HaCaT cells treated with PAO in the presence or absence of 4-PBA or NAC. (D) Immunofluorescence staining of spliced (s) XBP-1 in HaCaT cells treated with PAO in the presence or absence of 4-PBA or NAC. Arrows indicate nuclear localization of sXBP-1. Data are expressed as Mean ± SEM. *$P<0.05$, **$P<0.01$ and ***$P<0.001$ when compared to control. $^{\#}P<0.05$, $^{\#\#}P<0.01$ and $^{\#\#\#}P<0.001$ when compared to PAO.

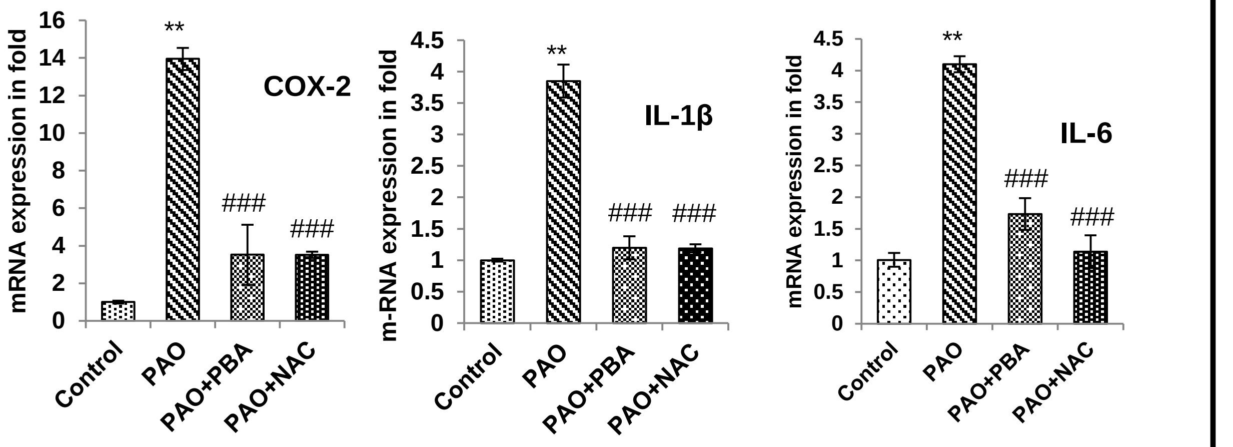

Supplementary Fig. S13: Real time PCR analysis of COX-2, IL-1β and IL-6 in HaCaT cells. **P<0.01 when compared to control. ###P<0.001 when compared to PAO.

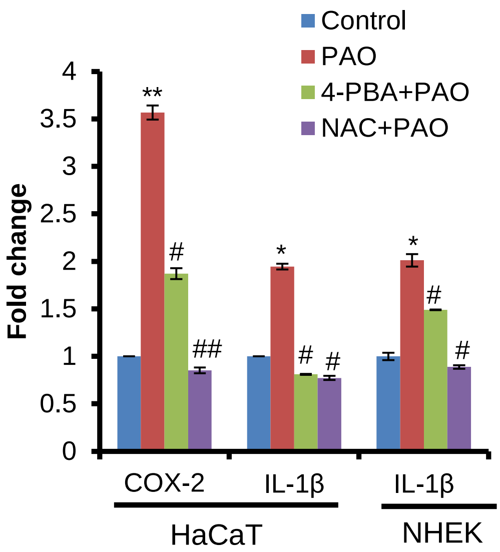

Supplementary Fig. S14: Dentiometric analysis of western blots for fig. 8E. β-actin was used as a loading control. *P<0.05, **P<0.01 when compared to control. #P<0.05, ##P<0.01 when compared to PAO-treated group.

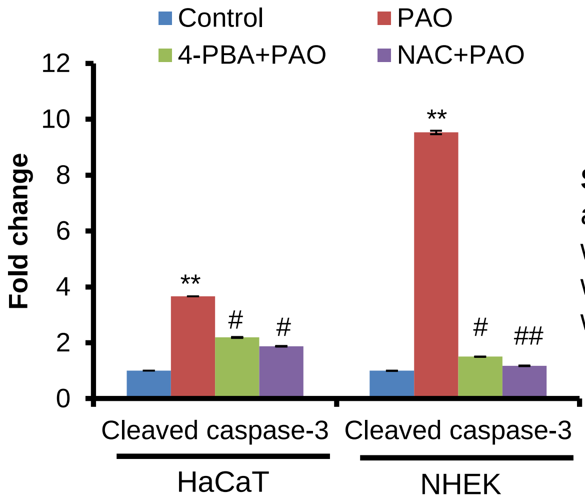

Supplementary Fig. S15: Dentiometric analysis of western blots for fig. 8F. β-actin was used as a loading control. **P<0.01 when compared to control. #P<0.05, ##P<0.01 when compared to PAO-treated group.

Supplementary Table- S1: PAO-induced skin changes in terms of clinical observation, erythema and edema.

**Draize Score**	**PAO-induced clinical observations**
0	Unchanged from age matched control site
1	Mild edema, mild erythema, or other mild changes from age matched control mice (no necrosis)
2	Moderate edema, moderate erythema, or other moderate changes from age matched control mice (no necrosis)
3	Severe edema, Severe erythema, or other Severe changes from age matched control mice (no necrosis)
4	Focal necrosis: focal area of tissue is necrotic
5	Mild necrosis: 25-50% of tissue is necrotic
6	Moderate necrosis: 50-75% of tissue is necrotic
7	Severe necrosis: 75-100% of tissue is necrotic

Supplementary Table- S2: List of primers used in this study.

**Real Time Primers (mouse)**	**Sequences**
IL-1β	F 5`-AAAGCCTCGTGCTGTCGGACC-3`
	R 5`-CAGGGTGGGTGTGCCGTCTT-3`
IL-6	F 5`-GGTGACAACCACGGCCTTCCC-3`
	R 5`-AAGCCTCCGACTTGTGAAGTGGT-3`
IFN-α	F 5-`CCCCTGACCCAGGAAGATGCC-3`
	R 5`-ACATTGGCAGAGGAAGACAGGGCTC-3`
TNF-α	F 5`-AGCCCACGTCGTAGCAAACCAC-3`
	R 5`-TCGGGGCAGCCTTGTCCCTT-3`
GAPDH	F 5'-CAATGTGTCCGTCGTGGATCT-3'
	R 5'-GTCCTCAGTGTAGCCCAAGATG-3'
**Real Time Primers (human)**	**Sequences**
Grp78	F 5`-GCCTGTGGCTGGACTGCCTG-3`
	F-5`-ACGCCGACGCAGGAGTAGGT-3`
Chop	F-5`-GGTGGCAGCGACAGAGCCAA-3`
	F-5`-CAGCTGCCATCTCTGCAGTTGGA-3`
sXBP-1	F-5`-GGTCTGCTGAGTCCGCAGCAGG-3`
	F-5`-GGGCTTGGTATATATGTGG-3`
GAPDH	F-5`-GGGGCTGGCATTGCCCTCAA-3`
	F-5`-GGCAGGGACTCCCCAGCAGT-3`
**TaqMan PCR Primers (human)**	**Cat. No. (ThermoFisher Scientific)**
COX-2	Hs00153133-m1
IL-6	Hs00985639-m1
IL-1β	Hs00174097_m1
TGF-β	Hs00998133-m1
GAPDH	Hs02758991-g1

Supplementary Table- S3: List of primary antibodies used in this study.
*IF- Immunofluorescence; **IHC- immunohistochemically

Antibody	Company	Application	Dilution
IL-1β	Abcam	Western blot	1000
COX-2	Cayman Chemicals	Western blot	1000
GRP78	Cell signaling/Santa Cruz	Western Blot/*IF	1000/200
p-PERK	Cell signaling	Western Blot	800
CHOP	Cell signaling	Western Blot/**IHC	1000/100
ATF6α	Santa Cruz	Western Blot	1000
p-eIf2α	Cell signaling	Western Blot	1000
ATF4	Abcam/Cell signaling	Western Blot/**IHC	1000/100
Cleaved caspase-3	Cell signaling	Western Blot	1000
